# Smoke Signals: Teasing Out Adverse Health Effects of Wildfire Emissions

**DOI:** 10.1289/ehp.124-A166

**Published:** 2016-09-01

**Authors:** Nancy Averett

**Affiliations:** Nancy Averett writes about science and the environment from Cincinnati, OH. Her work has been published in *Pacific Standard*, *Audubon*, *Discover*, *E/The Environmental Magazine*, and a variety of other publications.

Worldwide, the preponderance and severity of wildfires is increasing due to a number of factors, among them changes in temperature and precipitation patterns consistent with climate change.^1^ ^,^ ^2^ Aside from the acute threat of imminent death, wildfires expose people to a range of harmful pollutants in smoke.^3^ Although health effects are well documented for many of these individual pollutants—including carbon monoxide, nitrogen dioxide, particulate matter, and more—it is difficult to gauge the public health impact posed specifically by wildfire smoke inhalation. In a new review, investigators present evidence linking wildfire smoke exposures to increased overall mortality rates and multiple respiratory conditions.^4^

The authors reviewed 53 epidemiological studies that assessed mortality and morbidity outcomes in relation to wildfire smoke. They specifically targeted studies on respiratory, cardiovascular, mental, and perinatal health.

**Figure:**
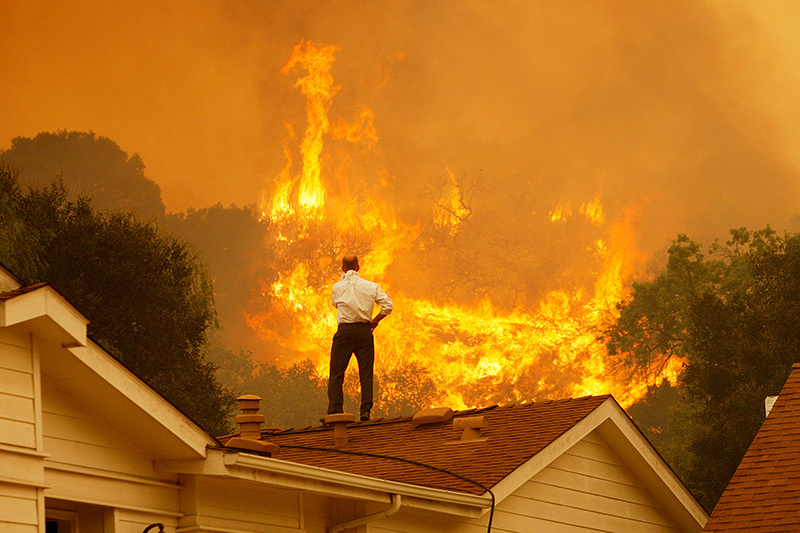
Springs Fire near Camarillo, California, 3 May 2013. A new review concludes there is strong evidence that wildfire smoke exacerbates asthma and COPD. © David McNew/Getty Images

“There is growing evidence of increased risk of all-cause mortality associated with smoke from wildfires,” says first author Colleen E. Reid, an environmental epidemiologist at Harvard University’s Center for Population and Development Studies. For example, each year, an average 339,000 premature deaths worldwide are estimated to result from wildfire smoke.^5^ “But,” she continues, “the evidence is not consistent for specific causes of mortality. So whether it’s respiratory or cardiovascular or even more specific causes of death, we don’t know.” Therefore, investigations involving larger populations are needed to better pinpoint specific causes of mortality.^4^

With respect to diseases resulting from wildfire smoke, Reid says there is very clear evidence of increased risk of exacerbated asthma and chronic obstructive pulmonary disease (COPD). “That alone is a finding worth noting,” says Kevin Tse, an allergist and immunologist at Kaiser Permanente Medical Center in San Diego, California, who was not involved in the review but has studied the effects of wildfire pollution on asthma. “Breathing issues,” he says, “can lead to significant morbidity and mortality, and at-risk populations might reconsider moving to an area where there is a high chance for wildfire exposure.”

Reid points out that several studies have also reported associations between wildfire pollution and increased respiratory infections, but again, further research is needed before investigators can establish a causal link. Likewise, the review found only inconclusive evidence for wildfire pollution’s effects on cardiovascular disease, mental health, and perinatal health.^4^

“There are a few studies that have found significant increases in out-of-hospital cardiac arrests, but most studies find null effects for other cardiovascular outcomes like hypertension or ischemic heart disease,” Reid says. “There are just too few studies, and the ones that have been done have not produced consistent results.”

The authors note that the strongest of the studies reviewed looked at very high-exposure events that lasted for longer periods, assessed larger populations over many years in areas with frequent fires, and utilized common indicators of health outcomes, such as the dispensation of asthma medicine.^4^

In choosing which studies to include, the authors assessed the risk of bias (i.e., systemic errors in the study design) for each study based on sample size, exposure assessment methods, control for confounding factors, and use of objective outcome measures.^4^ But bias in population studies can take a wide variety of forms that are impossible for researchers to control for despite their best efforts. “For example,” Tse says, “wind patterns on the days of the fires can cause significant scattering of PM_10_ and PM_2.5_ and affect areas quite distant from the wildfire epicenter. Also worth noting is that patients who are aware of their underlying asthma and COPD may have more quickly evacuated their homes at the onset of wildfire, thus not incurring significant morbidity and mortality during the worst parts of the fires.”

He adds, “As with most retrospective studies, researchers do the best with the data they have, but it is possible that there will continue to be many inconclusive results when studying wildfires because of these inherent, some would even say natural, biases.”

From the perspective of protecting public health, Reid and colleagues conclude that more information is needed before setting smoke thresholds that trigger public health warnings or interventions. For one thing, there needs to be a better understanding of the ways in which different levels of wildfire emissions affect health. It is also critical to consider which populations are most susceptible to health effects resulting from smoke exposure.^4^
